# Development of Brain EEG Connectivity across Early Childhood: Does Sleep Play a Role?

**DOI:** 10.3390/brainsci3041445

**Published:** 2013-11-12

**Authors:** Salome Kurth, Peter Achermann, Thomas Rusterholz, Monique K. LeBourgeois

**Affiliations:** 1 Sleep and Development Laboratory, Department of Integrative Physiology, University of Colorado Boulder, Boulder, CO 80309, USA; E-Mails: salome.kurth@colorado.edu (S.K.); t.rusterholz@pharma.uzh.ch (T.R.); 2 Institute of Pharmacology and Toxicology, Section of Chronobiology and Sleep Research, University of Zurich, 8057 Zurich, Switzerland; E-Mail: acherman@pharma.uzh.ch; 3 Zurich Center for Integrative Human Physiology, University of Zurich, 8057 Zurich, Switzerland; 4 Neuroscience Center Zurich, ETH and University of Zurich, 8057 Zurich, Switzerland

**Keywords:** sleep EEG, coherence, development, maturation, children, early childhood

## Abstract

Sleep has beneficial effects on brain function and learning, which are reflected in plastic changes in the cortex. Early childhood is a time of rapid maturation in fundamental skills—e.g., language, cognitive control, working memory—that are predictive of future functioning. Little is currently known about the interactions between sleep and brain maturation during this developmental period. We propose coherent electroencephalogram (EEG) activity during sleep may provide unique insight into maturational processes of functional brain connectivity. Longitudinal sleep EEG assessments were performed in eight healthy subjects at ages 2, 3 and 5 years. Sleep EEG coherence increased across development in a region- and frequency-specific manner. Moreover, although connectivity primarily decreased intra-hemispherically across a night of sleep, an inter-hemispheric overnight increase occurred in the frequency range of slow waves (0.8–2 Hz), theta (4.8–7.8 Hz) and sleep spindles (10–14 Hz), with connectivity changes of up to 20% across a night of sleep. These findings indicate sleep EEG coherence reflects processes of brain maturation—*i.e.*, programmed unfolding of neuronal networks—and moreover, sleep-related alterations of brain connectivity during the sensitive maturational window of early childhood.

## 1. Introduction

Across childhood and adolescence the brain undergoes massive morphological changes such as cortical refinement, synapse growth, pruning, and white matter myelination [1,2]. Driven by environmental and programmed factors, these processes cooperatively interact to optimize functioning of neuronal networks resulting in the maturation of simple and complex brain functions. However, how functional brain connectivity develops and the factors that moderate these processes are poorly understood. Coherence analysis reflects temporal correlations between remote neurophysiological events and thus, can be used to study the functional connectivity between brain regions [3]. Local voltage gradients obtained by bipolar derivations provide a quantitative estimate of the strength of the underlying local generators [3]. During development, major connectivity growth is assumed to contribute to neuronal network maturation, resulting in refinement of brain functions and behavioral skills. Sleep EEG coherence increases across adolescence [4], however, similar longitudinal research in early childhood is lacking. Here, we address whether sleep EEG-based connectivity changes during the preschool years and across a night of sleep.

Sleep is a key environmental contributor to brain optimization processes. Ocular dominance experiments on kittens demonstrate that sleep plays a crucial role in brain maturation [5]. For example, during the critical period of visual development, cortical plasticity is enhanced during sleep but not wakefulness. Moreover, cortical remodeling triggered by monocular deprivation was associated with the amount of non-rapid eye movement (non-REM) sleep during deprivation. In contrast, blocking neuronal activity during sleep via sodium channel blockers reduced plasticity [6]. Thus, in critical phases of development, the maturation of skills not only requires cortical activity during waking, but also a subsequent period of sleep. In humans, the well-established EEG marker of deep sleep, slow-wave activity (SWA, EEG power in the 1–4.5 Hz frequency range), undergoes maturation in parallel with cortical morphology [7,8] and might be interdependent with structural changes [9]. SWA or sleep spindles (10–14 Hz) seem to be involved in synaptic remodeling, leading to alterations in synaptic strength and synchronized neuronal firing [10,11,12]. The underlying cellular phenomenon of SWA is the slow oscillation [13,14], which is the alternation between depolarized (associated with neuronal firing) and hyperpolarized (associated with neuronal silence) membrane potentials. In deep sleep, slow oscillations are highly synchronized between neuronal units and concomitant with the positive or negative deflection of slow waves on the surface EEG [15,16]. SWA in particular has been proposed to reflect a renormalization of synapses that were potentiated during preceding waking, a process thought to rebalance synaptic homeostasis across a night of sleep [17]. Thus, through the interplay of cortical and thalamic interactions [18], the sequence of depolarization and hyperpolarization of slow oscillations likely promotes synaptic depression or elimination [10,11,19]. Thus, behavioral sleep and its characteristic globally synchronized activity could provide conditions for single-cell rest allowing uninterrupted hyperpolarized states [12].

The relationship between neuronal network maturation and brain activity (in wakefulness or sleep) is poorly understood. The structural backbone of the neuronal network entails cortical synapses and myelinated fiber tracts, which undergo massive modifications in the early years of life [1,2]. Considering the possible involvement of sleep in neuronal plasticity, it is conceivable that sleep is essential for such structural alterations. As the sleep EEG holds strong promise as an early indicator for predicting psychosocial and behavioral problems [20], it is fundamental to understand to what degree brain connectivity is mirrored in the sleep EEG, and whether maturation is facilitated by sleep itself. Although our analysis does not allow us to address this question, it represents an important first step in understanding early maturational changes in brain connectivity and the potential role of sleep.

In this study, we examined sleep EEG connectivity throughout childhood and the temporal dynamics across a night of sleep. Connectivity was assessed with sleep EEG coherence, which provides insight into temporal synchrony of distinct brain regions during sleep [3] and allows for the tracking of subtle dynamical changes of functional brain connectivity across development. EEG coherence is proposed to reflect white matter connectivity (myelination), while EEG power is thought to mirror gray matter (synaptic strength). Previous data allude to an increase of EEG coherence throughout adolescence [4]. Additionally, coherence is a measure of functional connectivity that is presumably driven primarily by fiber insulation, *i.e*., myelination. Because imaging data reveal a white matter myelin increase throughout childhood, we hypothesize a similar region-specific maturational increase in coherence. Additionally, recent findings indicate that neuronal activity during sleep preferentially impacts proliferation of oligodendrocyte precursor cells [21], the mediators of myelin formation. Indeed, if sleep plays a specific role in myelin production, we expect this process will be reflected in alterations of EEG connectivity. Thus, we tested the hypothesis that sleep EEG coherence changes across a night of sleep in early childhood.

## 2. Results and Discussion

**Figure 1:**
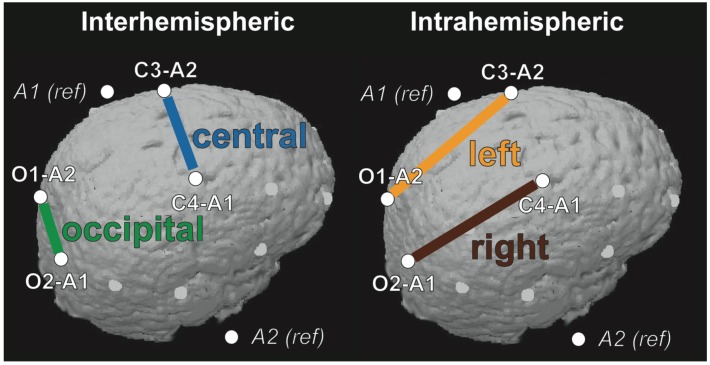
Coherence was derived from inter-hemispheric (**left panel**), central (C4A1-C3A2) and occipital (O2A1-O1A2) EEG derivations and from intra-hemispheric derivations (**right panel**) in the left (C3A2-O1A2) and right (C4A1-O2A1) hemisphere.

At three ages (2.5–3.0 years, “2Y”; 3.5–4.0 years, “3Y”; 5.5–6.0 years, “5Y”) one night of sleep (average duration: 606 ± 9 min at 2Y, 592 ± 10 min at 3Y, 581 ± 6 min at 5Y) was assessed in eight healthy children (3 males) following a stable sleep schedule. Standard sleep stage scoring [22] and artifact removal were performed before computing EEG coherence spectra of consecutive 30-s epochs (Hanning window, average of six 5-s epochs, Welch’s averaged periodogram method; see also Experimental Section): (a) inter-hemispheric coherence of *central* (C4A1-C3A2) and *occipital* (O2A1-O1A2) derivations in the two hemispheres; and (b) intra-hemispheric coherence between central and occipital derivations in the *left* (C3A2-O1A2) and *right* (C4A1-O2A1) hemisphere (Figure 1). Squared coherence between two simultaneously recorded EEG derivations as a function of frequency was calculated as shown in Equation (1):

*C_xy_*(*f*) = |*P_xy_*(*f*)|^2^/|*P_x_*(*f*)*P_y_*(*f*)| (1)

where *x* and *y* are the two signals *f* frequency, *P_x_*, *P_y_* power, and *P_xy_* cross-power spectra.

### 2.1. Connectivity Changes across Development

**Figure 2:**
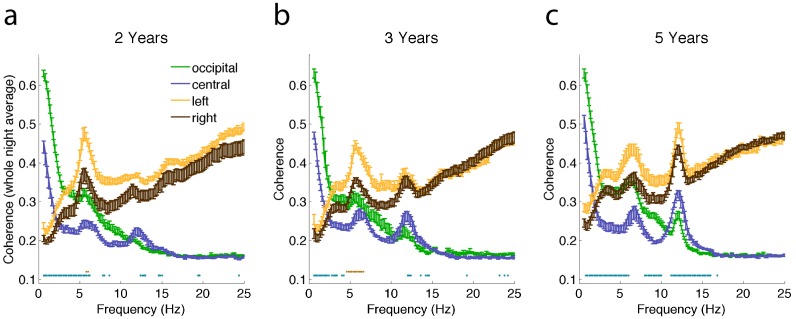
Average all-night coherence spectra [non-rapid eye movement (non-REM) sleep stages N2–N4, artifact free epochs only]. Dots refer to significant differences between inter-hemispheric coherence spectra (occipital *vs.* central, **green**), and intra-hemispheric coherence (left *vs.* right hemisphere, **brown**) using bootstrap statistics for each 0.2 Hz bin (*p* < 0.05). Fisher’s *z*-transformation was applied to the square root of coherence values for statistical analysis. Back-transformed, squared data are plotted (applies for all subsequent figures). (**a**) 2 Years; (**b**) 3 Years; and (**c**) 5 Years.

Inter-hemispheric sleep EEG coherence predominated in occipital compared to central areas across a broad frequency range below 10 Hz (Figure 2, repeated measures ANOVA including 0.6–25 Hz, *p* < 0.0005 “occipital” *vs.* “central” for 2Y, 3Y and 5Y). Intra-hemispheric coherence was larger in the left *vs.* the right hemisphere in the theta frequency range (Figure 2, *p* < 0.05 “left” *vs.* “right” for 2Y, 3Y and 5Y). Moreover, inter-hemispheric spectra showed high coherence in low frequencies and a decline with increasing frequency, while intra-hemispheric spectra showed an increase with faster frequencies. Visual inspection revealed increasing coherence in the spindle frequency range (10–14 Hz) with age, which may reflect the developmental change in sleep spindle length or density [23]. Based on differences between coherence derivations identified in the average spectral profile and oscillatory appearance of EEG activity (*i.e.*, “peaks” in the spectral profile), subsequent analyses were restricted to specific frequency bands: low-delta (0.8–2 Hz), theta (4.8–7.8 Hz) and spindle activity (10–14 Hz). The faster frequencies in the beta range (>20 Hz) exhibited high coherence in the intra-hemispheric derivations at all time points (Figure 2). Because only single 0.2 Hz bins exhibited across-night coherence alterations in the beta frequency range (as described subsequently), this frequency was not included for further analyses.

**Figure 3:**
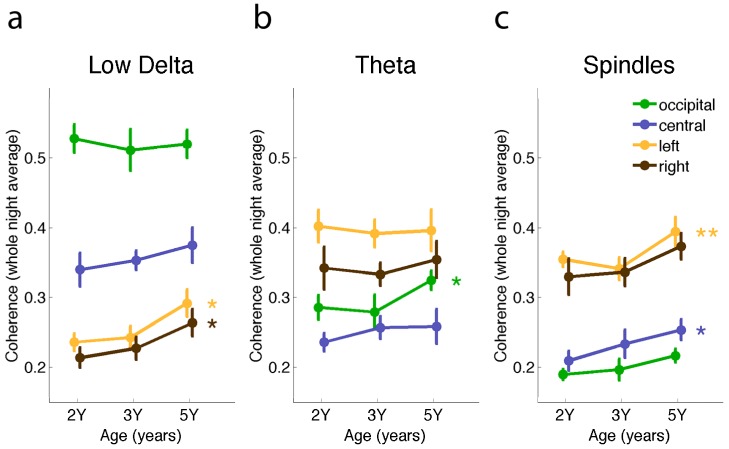
Development of sleep EEG coherence is region- and frequency-specific. Coherence data (inter-hemispheric “occipital” and “central”; intra-hemispheric “left” and “right”) were averaged for the whole night (mean ± SEM). Repeated measures ANOVA reached significance in the “left” and “right” hemisphere for low-delta coherence, inter-hemispheric “occipital” areas for theta and inter-hemispheric “central” areas and in the “left” hemisphere for spindle coherence, as indicated with asterisks (* *p* < 0.05; ** *p* < 0.01). *Post-hoc* two-tailed paired *t*-tests revealed developmental differences mainly between 2Y–5Y (see text for details). In subsequent analysis, frequency bands were defined as (**a**) low-delta (0.8–2 Hz); (**b**) theta (4.8–7.8 Hz); and (**c**) sleep spindles (10–14 Hz).

Coherence averaged in specific frequency bands showed developmental changes (Figure 3). Coherence either increased or remained constant across early childhood in the defined frequency bands; no developmental decrease was found in any derivation or frequency. We observed maturational changes in coherence in the low-delta range, namely an intra-hemispheric increase (Figure 3, repeated measures ANOVA, factor “age”). *Post hoc* paired *t*-tests showed that most differences prevailed between 2Y and 5Y, with low-delta activity in “left” (+14%) and “right” hemisphere (+14%, *p* < 0.005, percentage was calculated as 5Y relative to 2Y). In the theta frequency, coherence increased only inter-hemispherically in the occipital derivation (+9%, *p* < 0.05, 5Y/2Y). In the spindle frequency, coherent activity exhibited both intra- (left +7%) and inter-hemispherical (central +13%, *p* < 0.05, 5Y/2Y) increases. The intra-hemispheric left derivation also revealed a significant increase between 3Y and 5Y (11%, percentage calculated as 5Y/3Y) in the spindle frequency range.

### 2.2. Connectivity Changes across the Night

**Figure 4:**
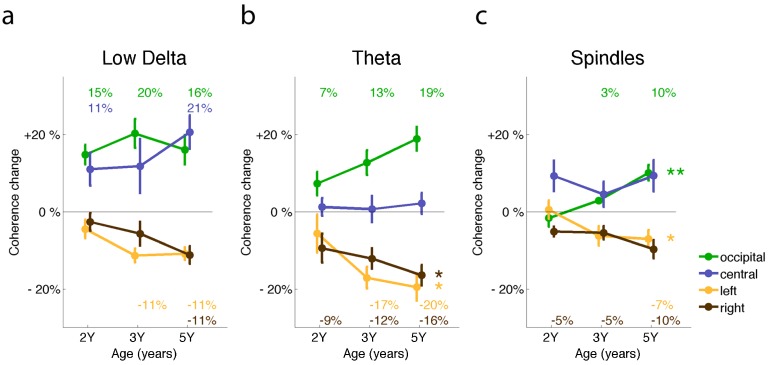
Temporal dynamics of coherence across the night presented for the frequency bands (**a**) low-delta (0.8–2 Hz), (**b**) theta (4.8–7.8 Hz) and (**c**) sleep spindles (10–14 Hz). First and last non-REM sleep episodes were compared (percentage change relative to first episode). Positive values reflect an increase across sleep, negative values a decrease (* *p* < 0.05; ** *p* < 0.01, repeated measures ANOVA, factor “age”). For data points that underwent a significant change across the night (*i.e.*, different from zero, *p* < 0.05, *post hoc* paired two-tailed *t*-test), the percentage of change is indicated in the corresponding color.

Next, we analyzed the temporal dynamics of connectivity across a night of sleep by comparing coherence across non-REM sleep episodes. Depending on frequency, derivation and age, coherence showed a sleep-dependent increase (positive % values; measured as % change of a given non-REM episode relative to the first episode), decrease (negative % values) or no change (0%). Overall, when a coherence change appeared across a night of sleep, a decrease was found in intra-hemispheric coherence in both hemispheres (most pronounced in theta range, Figure 4; results involving all non-REM episodes are provided in Supplementary Figures S1–S4), while an increase was detected in inter-hemispheric coherence in central and occipital areas. Quantifying the changes from the first to the last non-REM sleep episode, the maximal across-night coherence increase was found in occipital areas in the low-delta range, with increases up to +20% (2Y, Figure 4). In occipital but not central areas, coherence in the spindle frequency showed a greater across-night increase with age [“age”, *p* (occipital) < 0.01], reaching a maximum of +10% at 5Y. A decrease was observed for the theta range [“age”: *p* (left) and *p* (right) < 0.05]. Here, age was associated with a larger coherence decrease across the night [maximal reduction at 5Y of −20% (left) and −16% (right)]. No developmental changes in the low-delta range were observed in across-night coherence dynamics. However, the same analysis using a wider frequency range of delta (0.8–4.6 Hz) resulted in a developmental effect for the right intra-hemispheric derivation (*p* < 0.05, maximum of −8% at 5Y), showing a more pronounced across-night coherence decrease with increasing age.

### 2.3. Sleep EEG Coherence and Neuronal Networks

We observed developmental changes in not only sleep EEG coherence but also in across-night dynamics. Developmental dynamics of long-range connectivity across the early years of life support the growing evidence for sleep EEG features as promising markers for brain maturation [7,8,24,25]. Our longitudinal analysis helps to address the gap of knowledge on brain connectivity in early childhood and adds to prior findings showing maturational changes in EEG-based connectivity across adolescence [4]. Interestingly, we observed temporal coherence dynamics across a night of sleep. The coherence decrease in our data might be related to sleep homeostasis, which is well established and best reflected in SWA [26]. Homeostasis may reveal a sleep-dependent reduction of neuronal synchronization in the cortex [16]. In this study, coherence decreased only intra-hemispherically. It is conceivable that the main association fibers (superior and inferior longitudinal and the inferior fronto-occipital fasciculi) account for intra-hemispheric integration of cortical synchronization. Thus, our results suggest that intra-hemispheric cortical-subcortical integration networks experience an across-night weakening.

To our knowledge, an overnight functional connectivity increase has not been previously reported. This particular inter-hemispheric coherence increase implies a transitional stage of sleep-dependent strengthening of functional brain connectivity. Because such a finding has not been observed in adults [3], sleep-dependent connectivity dynamics might exclusively appear during early neuronal network maturation. Indeed, coherent brain rhythms represent a fundamental mechanism for sculpting temporal coordination of neural activity in brain-wide networks [27]. Relatedly, spike time dependent plasticity has been studied in the developmental context and refinement of developing circuits [28]. Thus, we believe that coherent sleep rhythms may serve to modulate brain networks as a process of brain maturation. Moreover, because coherence dynamics across a night of sleep seem to undergo age-specific modulations, they may thus be representative of sensitive maturational periods in childhood [5].

Oscillations of the sleeping brain are idiosyncratic. Spindles and slow waves are remarkably heritable [29], reveal trait-like stability within and across nights [30,31,32], and depend on white matter structure [33]. Slow waves and spindles are also affected by the preceding history of neuronal activation and synaptic plasticity [17,34]. Neuronal network dynamics are thus closely reflected in the sleep EEG. Even more, the main findings of this paper reveal developmental and across-night dynamics of coherence. Thus, EEG coherence might not only reflect the unfolding of neuronal networks, but might also emphasize sleep as a major process involved in brain maturation. An alternative explanation is that across-night strengthening (or weakening) appears as a result of an overall increase in the signal-to-noise-ratio. If so, a sleep-dependent rebalancing of synaptic strength mainly in the cortex [17] might appear as an increase of intrinsically solid connections. However, if this were true, one would expect this pattern to persist across all ages. Our data do not confirm this, but rather show age-dependent alterations in across-sleep dynamics. The intra-hemispheric coherence-decrease across a night of sleep might be related to the homeostatic decline of sleep propensity, thus, reflecting a decrease of network strength. Animal models are required to further investigate processes associated with sleep-dependent maturation of brain connectivity. The expression of connectivity-related genes may undergo transitional (age-dependent) modifications. Genes related to myelination, membrane synthesis and maintenance, such as myelin-associated oligodendrocyte basic protein (MOBP), myelin-associated protein (MAG) or plasmolipin (PPLP), which have higher mRNA levels during sleep than waking [35], may be expressed age-specifically and may be key factors for brain connectivity maturation.

Although our electrophysiological data do not demonstrate a direct impact of sleep on brain structure (as for example shown in mice using two-photon microscopy [9]), they show temporal dynamics of functional connectivity. Along with changed functional connectivity, effective connectivity—*i.e.*, the ability of the brain to process more efficiently—may also be altered. Indeed, memory performance and intelligence are associated with sleep spindles [34], as well as white matter connectivity [36,37]. Spindle coherence seems to reveal efficiency of information transfer in these networks and thus might reflect the integrity of thalamo-cortical networks. Because spindle activity changes over the course of development, it cannot be excluded that coherence changes are influenced by changes in spindle power.

### 2.4. Regional Variation in Coherence

Our results indicate that intra-hemispheric coherence is more pronounced in the left hemisphere (lateralized) in the low-delta and theta bands (*p* < 0.05, ANOVA), which is in line with earlier observations of a left connectivity dominance [38,39]. Additionally, a protracted development of the right hemispheric anterior-posterior connectivity was previously documented [40]. Neuronal substrates for a regional difference in growth rate might reflect inter-hemispheric differences in the superior longitudinal fasciculus, or a difference in cell size and axonal branching, as found in some Broca areas [41]. Thus, coherent EEG theta activity in the left hemisphere might subserve neuronal networks coupled to language development [39]. However, the underlying mechanism for theta specificity and its function are unclear at this time. Further, regional differences of occipital inter-hemispheric coherence exceed that of central coherence (*p* < 0.005, ANOVA, for low-delta, theta and spindle frequencies). We also found inter-hemispheric coherent theta activity in the occipital derivation changed across early childhood. This result suggests that neuronal network integration of the corpus callosum matures earlier for occipital than for central regions [42,43]. The corpus callosum is the largest white matter structure coordinating information between cerebral hemispheres [44], whose posterior regions are associated with sensory and motor integration [45]. Structurally, white matter (myelin) increase is greatest [46] and happens initially in the occipital cortex [1]. Early occipital maturation also holds for synaptic pruning [47,48]. Accordingly, processes contributing to occipital network efficiency experience a maturational refinement during the early years of life, while central networks are refined later. In line with this, we found a developmental trend (*p* = 0.07) in the across-night dynamics of theta oscillations reflecting high occipital network efficiency at early age, which may be related to the comparatively early maturation of the visual system [49].

Sleep spindle generation involves cortico-thalamic projections [50,51], which integrate thalamic and surrounding areas, internal and external capsules, and the corona radiata. The overall increase in coherent sleep spindle maturation is assumed to be reflective of these networks and their functional correlates such as cognitive performance [52]. Imaging data link cognitive processing to the lateral prefrontal and parietal cortex [53,54], which are in an anatomical network with the thalamus. Likewise, intra-hemispheric low-delta coherence is assumed to reflect neuronal networks related to the superior longitudinal fasciculus, the major fiber tract connecting anterior and posterior cortical areas. Indeed, functional connectivity between the hemispheres seems to mature prior to anterior-posterior connectivity, the latter not showing a marked increase until late childhood [55,56,57]. Relatedly, other EEG studies (in waking) show developmental trends in the anterior-posterior direction in terms of timing of EEG coherence increases across maturation [40], thus, supporting region-specific growth spurts. Together, these data indicate that coherence analysis is a valuable method for studying the development of functional coupling between brain structures involved in generating neural activities during sleep. However, understanding why specific frequency bands undergo different developmental and regional changes remains an important unanswered question.

### 2.5. Neuronal Network Refinement

Myelin development is considered the main contributor for overall maturation of brain connectivity and function [58]. Myelinated white matter [1] passively increases action potential propagation speed via fiber insulation. Although the cellular basis of learning has traditionally been understood to derive from synaptic modifications, myelin changes could also promote learning by improving synchrony of impulse transmission between cortical regions [59]. Recent findings show that the myelin sheath provides metabolic supplies for high neuronal activity [60], and action potentials can stimulate oligodendrocyte development and myelination [61] via signaling that does not require neurotransmitter release from synapses [59]. An action potential-induced glutamate release onto oligodendrocytes along axons has been suggested as a mechanism triggering myelin build-up [60,62,63,64]. It is conceivable that neuronal activity with a frequency typical for sleep slow oscillations could also induce myelin growth and enhance functional connectivity. Night by night, this increase might lead to structural changes observed in brain anatomy.

Although cortical activity and sleep are both essential for the development of brain functions [5,6], it is unclear whether sleep rhythms are actively involved in shaping the immature brain. While the mediating role of SWA has mainly been ascribed to synaptic changes in the cortex [17], myelin-induction triggered by neuronal activity would instead involve white matter—the subcortical backbone of cortical connectivity. Most recent animal work is supportive of sleep contributing myelin formation and maintenance [21].

Interestingly, gene transcripts specific to sleep involve, amongst others, membrane synthesis and myelin in particular [35,65]. Moreover, because fiber architecture is highly heritable [66] and thus programmed, the neuronal networks maturing during sensitive developmental windows could benefit from or even necessitate sleep. In other words, because sleep is not directly coupled to environmental input of learning and stimulation, it could provide a safe window in which “programmed” maturation processes take place. In turn, if sleep was disturbed, brain disorders in maturation may result [67]. Additionally, it has been suggested that fiber growth and synaptic maturation in the cortex are coupled mechanisms that trigger each other’s growth and maintenance [68]. Nonetheless, it remains speculative that during sensitive phases of brain development, sleep supports fiber growth and cortical development.

A consequence of programmed network adjustments is the refinement of brain function (language, sensory- and motor-integration, higher-order functions, *etc.*): all processes that experience well-documented developmental spurts across childhood [58]. Sleep-wake interactions with synaptic plasticity change with developmental stage, as documented in rats [9] and flies [69]. Further, an inter-relationship of disturbed sleep and developmental or mood disorders is conceivable [67]. A further disentanglement of brain maturation processes, including the role of sleep and its effect on brain function, is crucial. Together, the involvement of sleep in cortical and subcortical connectivity strength might fundamentally sculpt structural changes and play a role in sensitive periods of brain maturation.

## 3. Experimental Section

Eight healthy children (3 males; 6 Caucasians) were studied at three ages [2.8 ± 0.2 (2Y); 3.8 ± 0.2 (3Y); 5.9 ± 0.2 (5Y)] years [70]. Exclusion criteria were co-sleeping; bedtime/wake time schedule varying more than 2 h between weekdays and weekends; travel across more than two time zones within 3 months prior to study start; regular use of medication affecting sleep/daytime alertness/the circadian system; reported sleep problems; developmental disabilities, epilepsy, neurologic/metabolic disorders, chronic medical conditions, lead poisoning, or head injury involving loss of consciousness; conceptual age of <37 or >42 weeks; low birth weight; or family history (first degree) of diagnosed narcolepsy, psychosis or bipolar disorder. Of 78 children screened, 10 met the study criteria and 8 completed the longitudinal protocol. Incompletions were due to electrode skin sensitivity (*n* = 1) or study protocol noncompliance (*n* = 1). Families signed an institutional review board approved consent form. Parents were compensated with cash, and children received small non-monetary gifts throughout the protocol.

For the 5 days before the overnight sleep recording, children followed a strict sleep schedule in which they slept in their typical environment (home, daycare, family care) with a minimum sleep opportunity of 12.5 h (2Y and 3Y) or 12 h (5Y) per 24-h day. This schedule included a nap opportunity of at least 45 min at 2Y and at 3Y for the majority of the sample (*n* = 7; one child had stopped napping). The stabilization phase provided minimization of sleep restriction and entrainment of the circadian system. Compliance with the schedule was verified with wrist worn actigraphs, sleep diaries and daily reports of parents via email or telephone. Caffeine consumption and medication affecting sleep, alertness or the circadian system were prohibited during the study.

On the night following the stabilization phase, polysomnographic (PSG) all-night home sleep recordings were performed after 13 h of prior wakefulness (no napping; bed time 19:58 ± 0:11 (SEM) at 2Y, 20:05 ± 0:09 at 3Y, 19:59 ± 0:13 at 5Y; wake up time 6:47 ± 0:11 at 2Y, 6:52 ± 0:14 at 3Y, 6:34 ± 0:14 at 5Y) using a portable Vitaport 3 EEG recorder (Temec Instruments, Kerkrade, The Netherlands). Four EEG derivations (C3A2, C4A1, O1A2, O2A1; standard 10-20 system) were recorded. Sleep stages were visually scored for 30-s epochs (C3A2) according to standard criteria [22]. Power spectra of consecutive 30-s epochs (FFT, Hanning window, average of six 5-s epochs) were computed for the derivations C4A1, C3A2, O2A1, O1A2. Coherence spectra within (C3A2-O1A2; C4A1-O2A1) and between (C3A2-C4A1; O1A2-O2A1) hemispheres were computed for consecutive 30-s epochs using Welch’s averaged, modified periodogram method. Fisher’s *z*-transformation was applied to the square root of coherence for statistical analysis; back-transformed, squared data are plotted in the figures [3]. The frequency resolution was 0.2 Hz and frequencies up to 25 Hz were analyzed. EEG artifacts were removed semi-automatically. Epochs were excluded whenever power in the 20–40 Hz and 0.8–4.6 Hz band exceeded a threshold based on a moving average determined over twenty 30-s epochs. In two recordings with sweating artifacts, we excluded frequency bins up to 1.6 Hz, in one of these recordings additional artifacts were excluded in faster frequencies (7.6–8.6, 15.6–16.6, 22–24 Hz). In all spectral comparisons, the two lowest frequency bins (0.2–0.4 Hz) were excluded because of their sensitivity to low frequency artifacts. The first and last non-REM sleep episodes were compared whenever they reached a minimal duration of 10 min artifact-free non-REM sleep (stages N2–4). Average non-REM sleep episode duration and timing are provided in Table 1. 

**Table 1:** Average duration (min) and timing [midpoint of non-REM sleep episode relative to sleep onset, *i.e.*, first occurrence of sleep stage 2] of first and last non-REM sleep episodes included for comparison (mean ± SEM). Six to ten non-REM sleep episodes were completed across the whole sample, with an average of 8.6 ± 0.5 episodes at 2Y, 8.1 ± 0.4 at 3Y and 7.5 ± 0.3 at 5Y.

Age	Duration (min)		Timing (min after Sleep Onset)	
	*First*	*Last*	*First*	*Last*
2Y	66.8 ± 4.8	41.4 ± 5.6	33.4 ± 2.4	595.1 ± 8.1
3Y	69.2 ± 3.0	41.3 ± 4.5	34.6 ± 1.5	578.5 ± 20.4
5Y	76.9 ± 5.4	40.2 ± 4.5	38.5 ± 2.7	575.0 ± 9.5

Signal analysis and statistics were performed with the software package MATLAB (Mathworks, Natick, MA, USA) and the statistics toolbox (Mathworks). *Post hoc* two-tailed paired *t*-tests were performed if an ANOVA revealed a significant effect for “age” (2Y, 3Y and 5Y), “region” (left *vs.* right hemisphere; central *vs.* occipital derivations), or “across-night” (non-REM sleep episodes). For coherence spectra, bootstrap analyses were performed at each 0.2-Hz-frequency bin (using 5000 iterations). Bootstrap tests are well suited for EEG analysis because no assumptions for data distribution are required (see [4]). This statistic uses a random sample from the original data pool, and the false alarm rate is controlled [71]. Significance level for all tests was α = 0.05.

Some limitations of the present study should be considered. First, across-night temporal dynamics of EEG coherence may be related to brain connectivity alterations driven by the timing of the circadian clock. To explore the masking effects of the circadian system, we contrasted sleep states (*i.e.*, time spent in REM or non-REM sleep) and changes in coherence across the night. These analyses pointed to a possible relationship of time spent in REM sleep and coherence change across the night (data not shown), yet, these relationships were frequency and region-specific. The significance of different frequency bands and regions remains to be investigated.

We then examined if EEG power mainly contributed to the observed changes in EEG coherence. Correlations between power and coherence were performed using individual data points averaged for each non-REM sleep episode. Positive (mainly intra-hemispheric) and negative (inter-hemispheric) correlations were found in the low delta and theta frequency range, and a less clear pattern was observed in the sigma range (data not shown). Relatedly, SWA and spindle power increased in the course of preschool years, which might impact maturational changes in coherence spectra.

Second, the dropping of daytime naps is a natural maturational shift during early childhood, and thus, a difficult factor to control during the stabilization segment of the study. At 2Y, all children napped on a regular basis, and at 3Y, one child had given up napping. By 5Y, none of the children in this longitudinal study napped. At all assessments, naps were not allowed on the day of sleep recordings, which likely resulted in increased sleep pressure, particularly in younger participants. These differences could technically have influenced our findings; however, one would expect an impact on EEG power rather than on coherence.

## 4. Conclusions

Reflecting the programmed unfolding of neuronal networks, coherence is a strong proxy for maturation of brain connectivity. Moreover, sleep may fulfill a central role in the development of fibers yielding to optimized network functioning. Consequently, when brain regions and related skills undergo maturation during sensitive windows, they might be particularly vulnerable to sleep disturbances.

## Supplementary Files

Supplementary File 1 Supplementary Information (PDF, 313 KB)
