# Supplementary material for: Development of Brain EEG Connectivity across Early Childhood: Does Sleep Play a Role?
Source: Brain Sci. 2013 Nov 12;3(4):1445–60. doi: 10.3390/brainsci3041445 (PMC3925344; doi:10.3390/brainsci3041445)
Supplement: Supplementary File 1 — Supplementary Information (PDF, 313 KB) [file brainsci-03-01445-s001.pdf]

Supplementary Information

Figure S1. Temporal dynamics of inter-hemispheric occipital coherence across the night. ***Left column***: Average coherence spectra as percentage change of last relative to the first non-REM sleep episode (last non-REM sleep episode was determined individually in each subject and ranged from the 6th to 10th non-REM sleep episode, see Table 1). For last non-REM sleep episodes, only data were included which contained at least twenty 30-s epochs. Significant changes across the night are represented as dots at the bottom of the figure (bootstrap statistics, 0.2-Hz frequency resolution). Red dots refer to significant increases in coherence from the first to the last non-REM episode and black dots refer to significant decreases ($p < 0.05$). ***Right column***: Temporal evolution of coherence across the night representing all non-REM sleep episodes (numbered). Spectra are expressed as a percentage change from the first non-REM sleep episode. Significant coherence changes across the night are represented as red (coherence increase) or black dots (decrease, $p < 0.05$, repeated measures ANOVA, factor "non-REM sleep episode"). Data are shown for the maximal number of non-REM sleep episodes completed by all subjects.

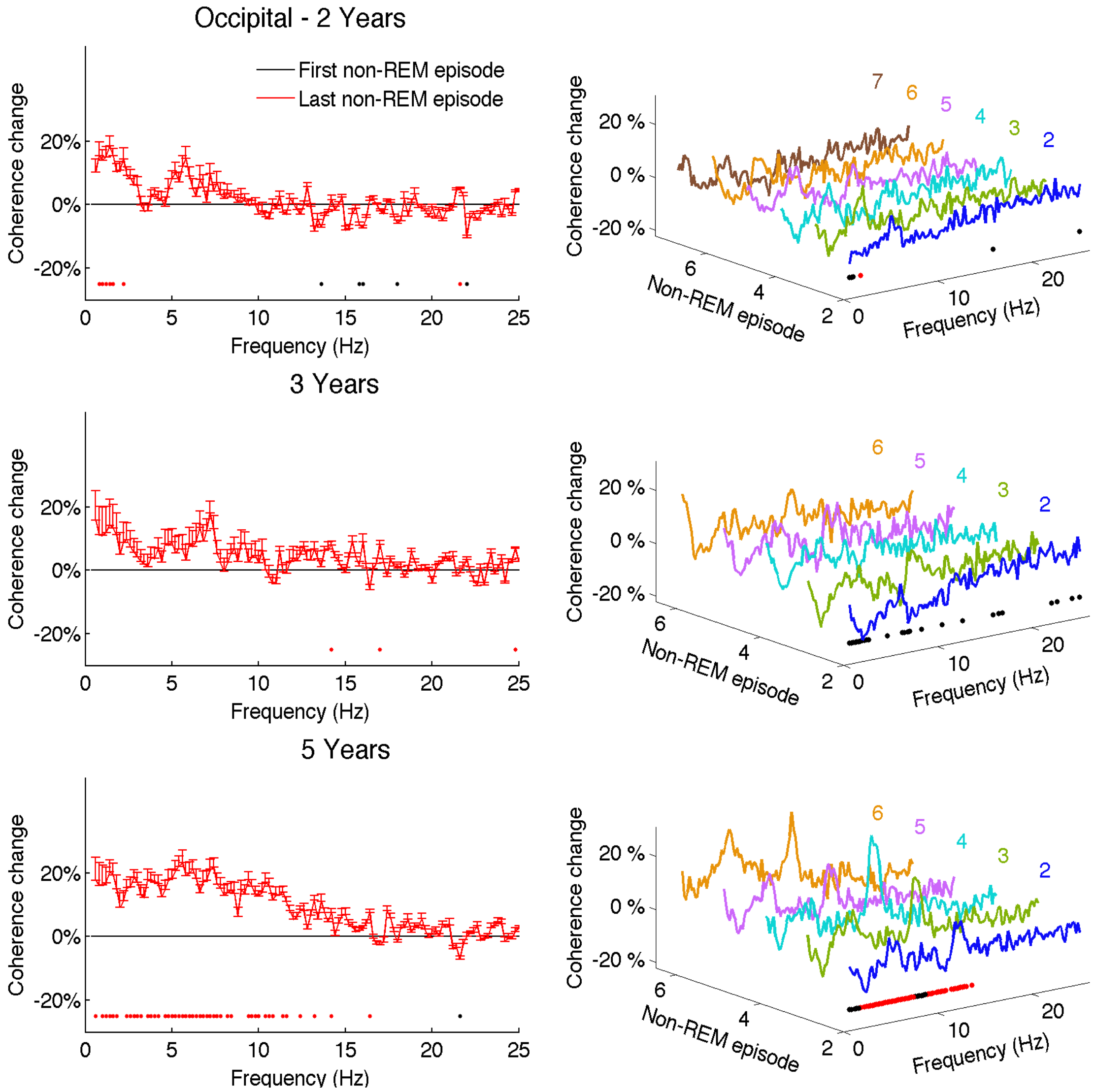

Figure S2. Temporal dynamics of inter-hemispheric central coherence across the night. For details see Figure S1.

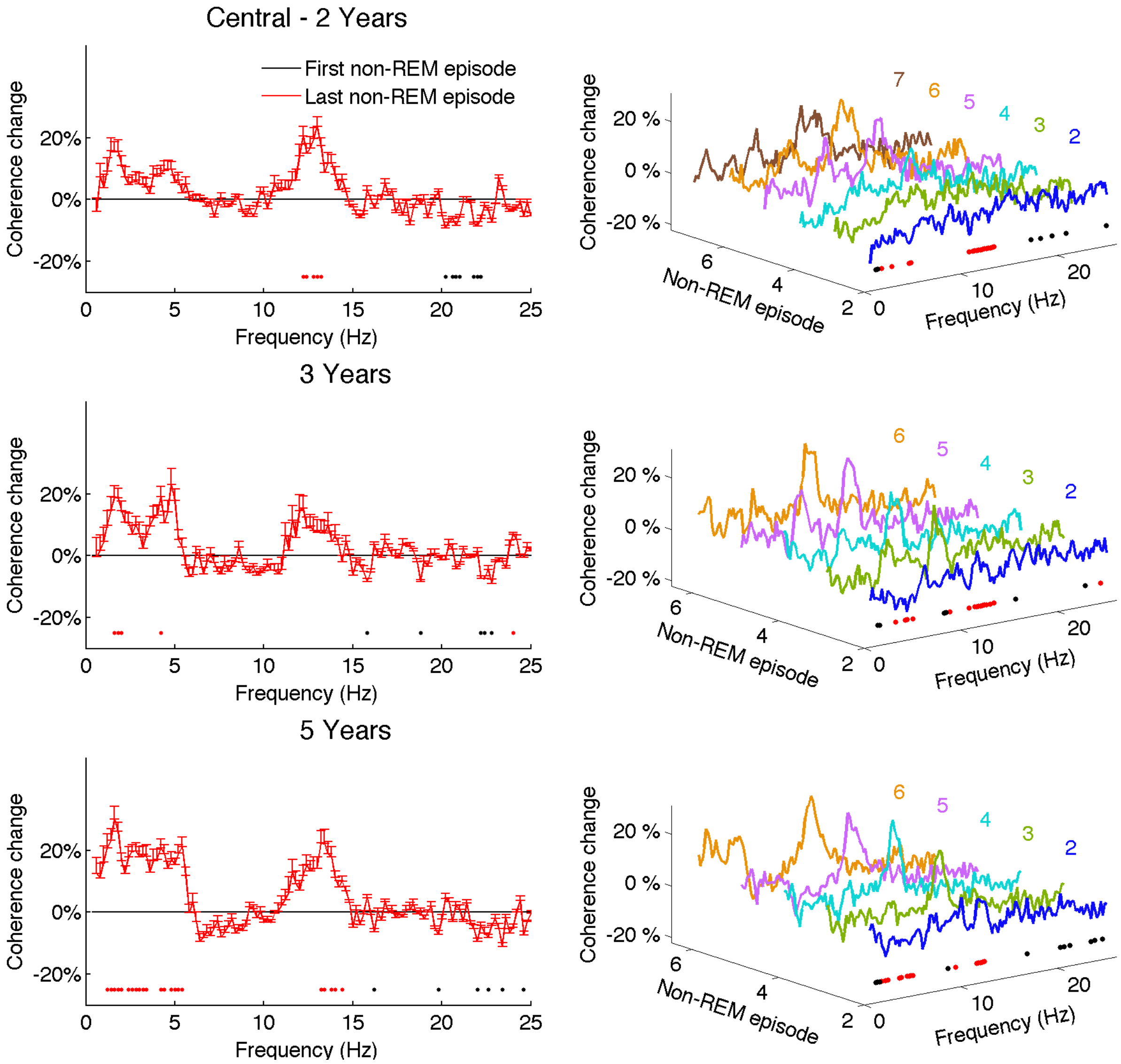

Figure S3. Temporal dynamics of intra-hemispheric coherence in the left hemisphere across the night. For details see Figure S1.

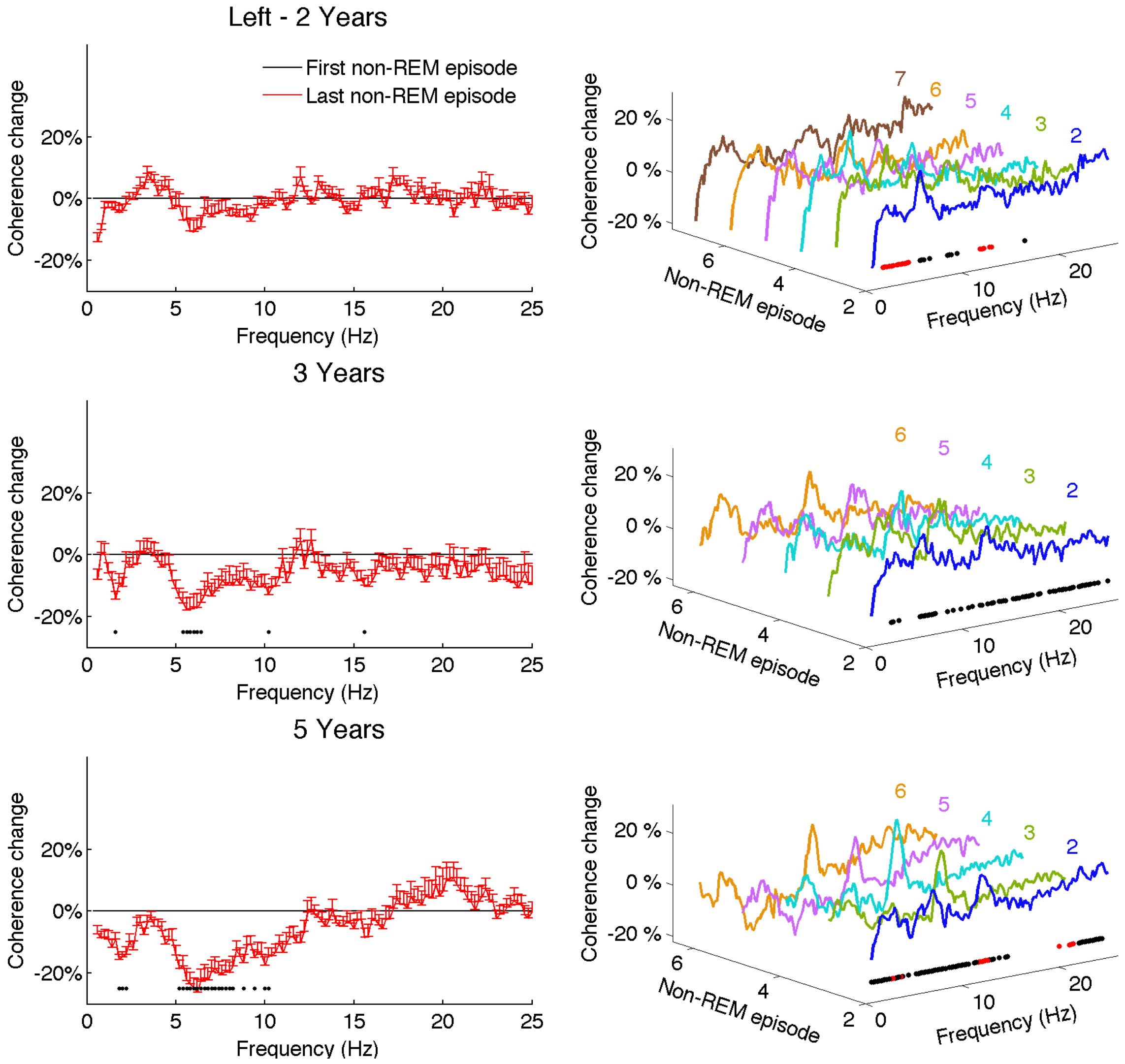

Figure S4. Temporal dynamics of intra-hemispheric coherence in the right hemisphere across the night. For details see Figure S1.

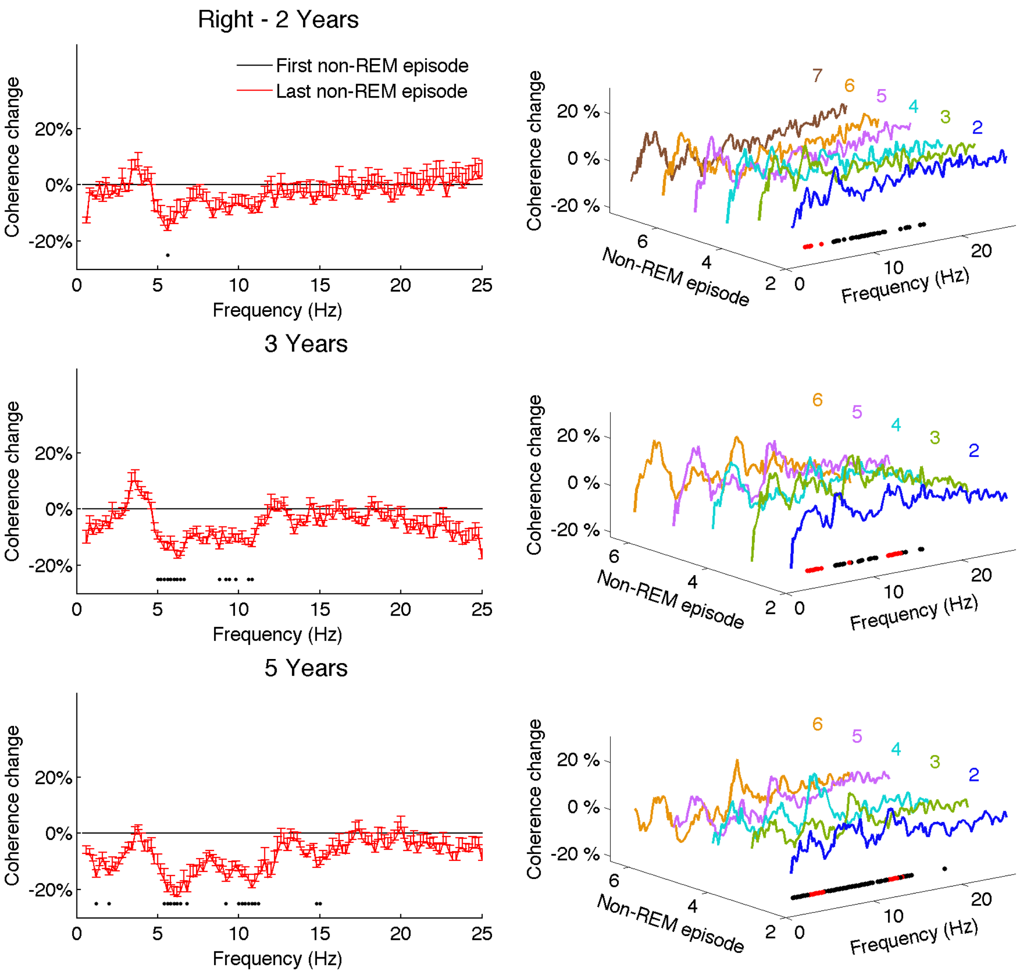

© 2013 by the authors; licensee MDPI, Basel, Switzerland. This article is an open access article distributed under the terms and conditions of the Creative Commons Attribution license (http://creativecommons.org/licenses/by/3.0/).
